# Integrated Pathways of COX-2 and mTOR: Roles in Cell Sensing and Alzheimer’s Disease

**DOI:** 10.3389/fnins.2020.00693

**Published:** 2020-07-09

**Authors:** Arti Tyagi, Mohammad A. Kamal, Nitesh Kumar Poddar

**Affiliations:** ^1^Department of Biochemical Engineering and Biotechnology, Indian Institute of Technology Delhi, New Delhi, India; ^2^King Fahad Medical Research Center, King Abdulaziz University, Jeddah, Saudi Arabia; ^3^Enzymoics, Hebersham, NSW, Australia; ^4^Department of Biosciences, Manipal University Jaipur, Jaipur, India

**Keywords:** COX, neuro-inflammatory, Alzheimer’s, mTOR, cellular pathways

## Abstract

Cyclooxygenases (COX) are enzymes catalyzing arachidonic acid into prostanoids. COX exists in three isoforms: COX-1, 2, and 3. COX-1 and COX-2 have been widely studied in order to explore and understand their involvement in Alzheimer’s disease (AD), a progressive neuroinflammatory dementia. COX-2 was traditionally viewed to be expressed only under pathological conditions and to have detrimental effects in AD pathophysiology and neurodegeneration. However, an increasing number of reports point to much more complex roles of COX-2 in AD. Mammalian/mechanistic target of rapamycin (mTOR) has been considered as a hub which integrates multiple signaling cascades, some of which are also involved in AD progression. COX-2 and mTOR are both involved in environmental sensing, growth, and metabolic processes of the cell. They are also known to act in cooperation in many different cancers and thus, their role together in normal cellular functions as well as AD has been explored in this review. Some of the therapeutic approaches targeting COX-2 and mTOR in AD and cancer are also discussed.

## Cyclooxygenase and Its Isoforms

Cyclooxygenase-2 (COX-2) is an isoform of the cyclooxygenase enzyme family, along with COX-1 and COX-3, which are involved in the synthesis of prostanoids (eicosanoid sub-class) from an essential fatty acid, namely arachidonic acid (AA). AA is first released by the action of phospholipase A2 (PLA2) from plasma membranephospholipids, which acts at the sn-2 position of the phospholipid backbone (Engelking, 2015; Hanna and Hafez, 2018). PLA2 activity is stimulated by microbial products, thrombin, immunoglobulins, etc., whereas anti-inflammatory glucocorticoids are known to inhibit it (Engelking, 2015). Out of the 20 + different types of PLA2s known to occur, type IIA secretory PLA2 and type IV cytosolic PLA2α have been shown to be coupled with COX-2 in different cell types. These two subclasses of enzymes have also exhibited cross-talk amongst themselves (Murakami et al., 1997; Balsinde et al., 1998; Bidgood et al., 2000; Sun et al., 2010). However, there are views that sufficient and conclusive data is still not present to establish a clear relationship between secretory PLA2 and eicosanoid signaling (Burke and Dennis, 2009).

**FIGURE 1:**
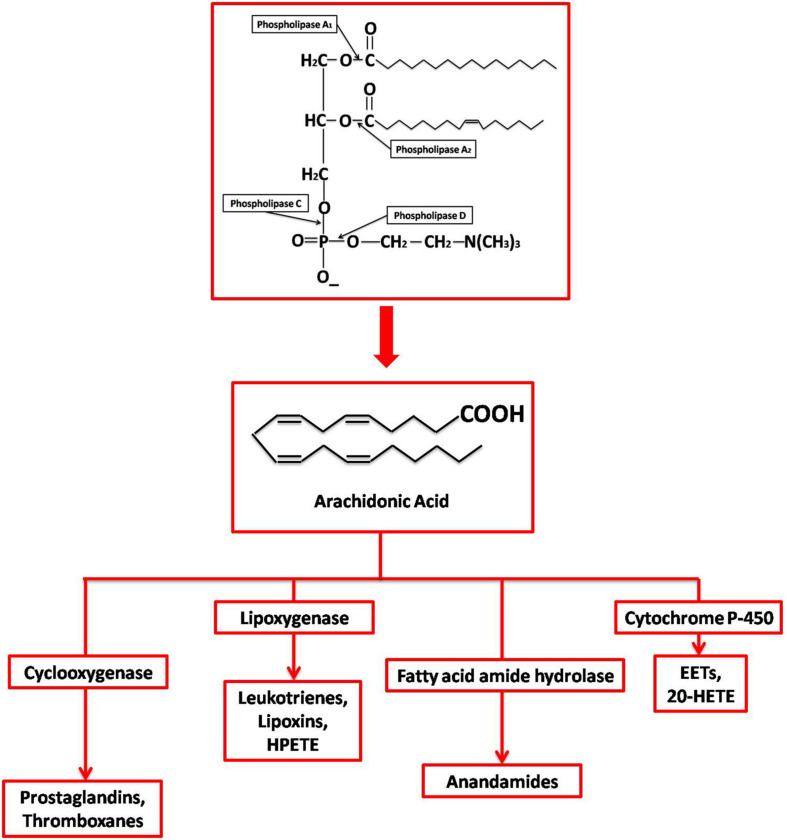
Synthesis and metabolization of Arachidonic Acid. Different phospholipases act at different sites on membrane phospholipids generating various products, including free AA by PLA2. AA is then enzymatically metabolized by (1) Cyclooxygenases into prostaglandins; (2) Lipoxygenases into leukotrienes, lipoxins, and 8- 12- 15-hydroperoxyeicosatetraenoic acid (HPETE); (3) Cyctochrome P450 into epoxyeicosatrienoic acid (EET) and 20-hydroxyeicosatetraenoic acid (20-HETE); and (4) Fatty acid amide hydrolase into endocannabinoid and anadamide.

Once AA is released, it is further metabolized by either enzymatic or non-enzymatic processes. Four different enzymatic pathways can act on AA: cyclooxygenase, lipoxygenase, anandamide, and cytochrome p450, resulting in different kinds of eicosanoids (Figure 1). Both COX-1 and COX-2 are part of the cyclooxygenase pathway, giving rise to prostanoids such as prostaglandin (PG) E_2_, PGF_2α_, PGD_2_, PGI_2_ (prostacyclin), and thromboxane (TX) A2. Prostanoids are a part of vasoactive lipids that act as local hormones and play crucial roles in normal physiology as well as certain pathophysiological states. The COX enzymes were popularized in 1971 upon demonstrating that non-steroidal anti-inflammatory drugs (NSAIDs) exert their potent anti-inflammatory properties via inhibition of COX. Since then, a lot of research has been dedicated to this unique enzyme and the various roles it plays in human physiology. Now, it has been established that NSAIDs also affect many other molecules, but COX remains an important player in various conditions, including certain neuropathologies. Some of these pathologies are also found to be epigenetically regulated as faulty DNA hypermethylation causes transcriptional silencing of COX-2 gene (Kikuchi et al., 2002; Ma et al., 2004).

COX is an integral membrane glycoprotein that carries out the first two committed steps of the prostanoid synthesis pathway: cyclooxygenation and peroxidation. Thus, it’s a bi-functional enzyme with a homodimeric structure and a single heme, involved in both the catalytic steps, situated in the middle of the two active sites of the enzyme (Rouzer and Marnett, 2009). In cyclooxygenation, two oxygen molecules are added to AA, forming the cyclic PGG2 (Prostaglandin G2) and later PGH2 (Prostaglandin H2) in the peroxidation step. PGH2 is an unstable intermediate that is acted upon by specific synthases and isomerases giving rise to different PGs and TX in a cell-specific manner (Clària, 2003). The COX isoforms are encoded on different chromosomes and have a lot of differences in their structural and regulatory organization, although they share a 60% amino acid sequence homology in their respective proteins (Clària, 2003). COX-2 gene has many regulatory sequences, like a TATA box, two NF-κB sites, a NF-IL6 motif, a CRE motif, and an E box, among others, which are absent in COX-1 gene (Clària, 2003; Kang et al., 2007). Many studies have been conducted in this regard; for example, CREB and Ets family-proteins (Ets-1 and Elk-1) were found to upregulate COX-2 expression in pancreatic β-cells. A detailed account of the different transcriptional factors involved in COX-2 regulation in different cell types has been given by Kang et al. (2007). This is keeping in line with the fact that COX-1 is generally constitutively expressed (Minghetti, 2004; Hoozemans et al., 2008), whereas COX-2 is induced in response to inflammatory reactions (Clària, 2003). Thus, COX-1 is often associated with PG synthesis under normal physiological conditions leading to phenomena like platelet aggregation, renal perfusion maintenance, and gastric cytoprotection. However, it has been found that COX-2 is also expressed in the kidneys, brain, and testes under physiological conditions along with COX-1 (Minghetti, 2004; Hoozemans et al., 2008). Specifically, in the brain, COX-2 has been proposed to influence memory, sensory integration, and autonomic regulation in the central nervous system (CNS) (Kaufmann et al., 1997). COX-3, on the other hand, is most populated in the cerebral cortex region of the brain and its enzymatic activity is inhibited by acetaminophen (Minghetti, 2004). However, much remains to be done to completely understand the functioning of COX-3. Both COX-2 and COX-1 are sources of pro-inflammatory prostanoids (Smyth et al., 2009); however, new evidence is fast emerging that also points to their role in resolution of inflammation (Williams et al., 1999; Fukunaga et al., 2005; Maskrey et al., 2011). Resolution is a complex process that kicks in once a sufficient inflammatory response has been mounted at the site of inflammation and is triggered by many cellular and molecular cues, such as macrophagic ingestion of apoptotic neutrophils (Ortega-Gómez et al., 2013).

## Mammalian/Mechanistic Target of Rapamycin (mTOR)

Rapamycin is a bacterial compound with anti-fungal, anti-cancer, and immunosuppressive properties, and thus, its target molecule, mTOR (mammalian/mechanistic target of rapamycin) has been implicated in processes such as aging, autophagy, and immune responses (Iglesias-Bartolome et al., 2012; Saxton and Sabatini, 2017) along with cellular pathways of proliferation, transcription, and translation (Shafei et al., 2017). The mTOR protein forms two complexes called mTORC1 and mTORC2, with a set of adaptor proteins. mTORC1 and mTORC2 are involved in different regulatory pathways and have different functions. The upstream regulatory components of mTOR include AMP-activated protein kinase (AMPK), glycogen synthase kinase (GSK3), insulin/insulin-like growth factor 1 (IGF-1), and phosphoinositide 3-kinase (PI3-K)/protein kinase B (Akt) while some of the major downstream regulators are 4E-binding proteins (4EBPs) and ribosomal protein S kinase (S6K) (Mueed et al., 2019; Papadopoli et al., 2019).

## COX and Alzheimer’s Disease

A lot of work has especially been dedicated to studying the role of COX-2 in neuroinflammatory and neurodegenerative conditions as its overexpression has been related to some of these pathologies (Minghetti, 2004). One of the prime targets of PGs includes the central and peripheral nervous system. The expression of COX-2 in certain types of mammalian neurons is “dynamically regulated” (Minghetti, 2004) by the synaptic activity of these neurons. Many *in vitro* as well as animal model studies have implicated the role of COX-2 in normal synaptic activity and plasticity (Hölscher, 1995; Kaufmann et al., 1997; Bazan, 2003), yet mice that had been genetically manipulated to knockout COX-2 gene exhibited a more or less normally functioning brain. However, in all these cases, accurate behavioral studies could not be performed as the mice suffered from renal failure very early in their lifespan (Dinchuk et al., 1995; Minghetti, 2004).

Alzheimer’s disease (AD) is a progressive neurodegenerative disease and one of the most prevalent types of dementia worldwide (Mueed et al., 2019). It is pathologically marked by the presence of two specific types of deposits in the brain: β-amyloid plaques and neurofibrillary tangles (NFTs), leading to neuroinflammation, synaptic dysfunction, and eventually neurodegeneration. NFTs are mainly composed of hyperphosphorylated tau (a microtubule associated protein) (Grundke-Iqbal et al., 1986; Alonso et al., 2001) while β-amyloid plaques are formed due to self-aggregation of the hydrophobic Aβ peptide produced upon cleavage of amyloid precursor protein (APP) (Luo et al., 2016; Mueed et al., 2019). The neuronal autophagy-lysosomal system is affected in AD pathology and results in low clearance of misfolded and damaged proteins, leading to aggregate formation (Shacka et al., 2008).

Epidemiological studies conducted on AD showed that populations which had a long history of NSAID use, were at a lower risk of AD (Breitner, 1996; Aisen, 2002; Minghetti, 2004; Hoozemans et al., 2008). This, along with other studies showing associations between COX-2 induction and neurodegeneration (Miettinen et al., 1997) as well as brain parenchymal amyloid plaque formation (Stewart et al., 1997), sparked interest, and subsequent research on potential effects of the COX enzymes and their various pathways in AD progression as NSAIDs are known inhibitors of COX.

Additionally, it has also been shown that lipids and PGs play vital roles in AD (Bazan et al., 2002; Thomas et al., 2016). AA, which gives rise to PGs, also happens to be the second most abundant polyunsaturated fatty acid in the brain (Thomas et al., 2016). It was also found that free AA affects synaptic functions of the brain (Latham et al., 2007). Thus, the levels of free AA in the brain cells, along with a balance between enzymes converting it to other molecules like PGs and enzymes releasing free AA, is also an important factor in AD (Thomas et al., 2016). In spite of this buzz about involvement of COX in AD, clinical studies carried out with NSAIDs and selective COX-2 inhibitors did not show any significant effects in treatment of AD (Hoozemans et al., 2008). Thus, it can be inferred that the effects of COX enzymes are not as straightforward in the case of AD as previously thought and the exact mechanisms by which they influence AD progression are not yet known.

The amyloid plaques, a classical hallmark of AD, are associated with pro-inflammatory cells and proteins. These proteins are found in the brain throughout the various stages of AD progression (Hoozemans et al., 2008). It is also speculated that inflammatory molecules may have dual role in AD as they exert both beneficial as well as detrimental effects based on their concentration and the stage of AD in which they are being expressed (Hoozemans et al., 2008). Along with this, epidemiological and genetic evidence also exist which points to the fact that inflammation is one of the key processes contributing to AD. However, due to failure of NSAIDs and selective COX-2 inhibitors against AD, alternate views are being considered which think of these drugs to perhaps have a preventive role with respect to AD and inflammation as merely a secondary phenomenon to clear off debris generated from more central underlying processes of AD (Wyss-Coray and Mucke, 2002; Hoozemans et al., 2006). It has also been reported that COX-2 expression in AD brains is correlated with altered expression of cell cycle proteins (Hoozemans et al., 2008). It was found that cell proliferation, adhesion, and differentiation genes were some of the most commonly upregulated ones along with PG synthesis genes in AD brains (Blalock et al., 2004).

COX-2 is constitutively expressed in certain cell populations of the brain, and additionally it is induced by inflammatory molecules such as cytokines, IL-1, IL-2, and TNF-α (Hoozemans et al., 2008). But, it is expressed in microglia (the local macrophages of the brain) only under very specific conditions, such as in chronic cases of cerebral ischemia (Tomimoto et al., 2000; Walsh et al., 2000). The expression of COX-2 is also absent in astrocytes (Pasinetti and Aisen, 1998; Hoozemans et al., 2001). COX-2 expresses differentially in different stages of AD and has pleiotropic functions in the brain (Hoozemans et al., 2008). In the early stages of the disease, COX-2 expression is increased (Ho et al., 2001; Hoozemans et al., 2001, 2002) and it is primarily expressed in pyramidal neurons (Braak and Braak, 1991). IL-1 levels also go up in AD due to association of neuritic plaques with microglial cells that express IL-1 (Blum-Degena et al., 1995). It is then reasonable to think that these IL-1 molecules perhaps induce higher expressions of COX-2. The expression of COX-2 has also been linked to cell cycle control (Xiang et al., 2002; Wu Chen et al., 2004), which may be involved in (re)generative pathways. However, these reasons are still controversial as the elevated expressions of COX-2 occur before the activation of microglia and astrocytes as shown by neuropathological studies (Hoozemans et al., 2002), whereas the expression has been found to reduce later on as the disease progresses and the number of neurons expressing COX-2 in severe cases of AD is very few (Hoozemans et al., 2004), which might be due to selective degeneration of neurons at these stages and a loss in the synaptic activity (Hoozemans et al., 2008).

It has even been observed that NSAIDs affected molecules and pathways involved in AD other than COX; for example peroxisome proliferator activated receptor γ (PPARγ) is found to be up-regulated in AD and can be activated by NSAIDs (Kitamura et al., 1999). Activation of PPARγ leads to clearance of Aβ deposits in neuronal as well as non-neuronal cells (Camacho et al., 2004). NSAIDs have also been found to lower Aβ deposits by targeting the γ-secretase enzyme and inhibition of nuclear factor-κB (NF-κB) pathway (Weggen et al., 2001; Morihara et al., 2002; Eriksen et al., 2003).

## Role of mTOR in AD

Recently, mTOR has also been found to be associated with AD pathology. Since AD is an age-associated disease, classical signs of aging like loss of proteostasis, mitochondrial dysfunction, altered intercellular communication, etc. can also be associated with mTOR (López-otín et al., 2013; Papadopoli et al., 2019). Since mTOR hinders autophagy, it promotes Aβ deposition in brain (Uddin et al., 2018). Due to disruption of the autophagic-lysosomal pathway, immature autophagolysosomes give rise to autophagic vacuoles that are then populated by β-amyloid plaques (Mueed et al., 2019). Rapamycin, however, is known to lessen these numbers (Haung Yu et al., 2005). Normally, adults contain very few autophagic vacuoles in their neurons (Cuervo et al., 2005) while these go up significantly in the affected regions of an AD brain (Haung Yu et al., 2005). An opposite connection has also been reported where induction with Aβ activates mTOR and its regulatory components (Bhaskar et al., 2009). Studies in both mouse models and humans have shown that the mTOR pathway is hyperactivated in AD (Perluigi et al., 2015). However, it has also been suggested that different neurons in an AD brain may have either upregulated or downregulated signaling cascades of mTOR based on how they react to AD stresses (Pei and Hugon, 2008).

Protein misfolding and subsequent aggregation is at the heart of Alzheimer’s pathophysiology (Mueed et al., 2019). The mTORC1 complex has been reported to stimulate protein synthesis (Papadopoli et al., 2019). However, there have been contradicting reports in case of regulation of proteolysis, with some claims of mTOR suppressing protein degradation (Zhao et al., 2015; Rousseau and Bertolotti, 2016) and others of mTOR enhancing it (Papadopoli et al., 2019). Thus, mTOR can play a role in the aberrant proteostasis in AD patients. Tau is one of the proteins whose translational pathway involves mTOR-dependent signaling via a 5’top mRNA (Pei and Hugon, 2008). Thus, upregulated and continuous production of tau proteins in degenerating neurons is regulated by mTOR signaling via the P70S6K and 4EBP1 pathways (Pei et al., 2008).

Moreover, mTOR mediates tau synthesis and phosphorylation at specific sites and is found to co-localize with NFTs (An et al., 2003; Tang et al., 2013). This results in microtubule instability as tau is an essential microtubule associated protein and its hyperphosphorylation causes it to detach from microtubules (Mueed et al., 2019).

## COX-2 and mTOR in Cell Metabolism and Signaling Cascades: Implications in AD

Small metabolites are known to be able to cross the blood-brain barrier and are also affected by environmental and genetic cues. Aberrant levels of amino acids, lipids, certain neurotransmitters, and other metabolites are found in both blood and brain in mouse models of AD (Pan et al., 2016). Many phospholipids have also been demonstrated to contribute to AD occurrence and progression (Li D. et al., 2016). Additionally, a general loss of protein homeostasis and glucose metabolism are characteristic of AD (Papadopoli et al., 2019).

**FIGURE 2:**
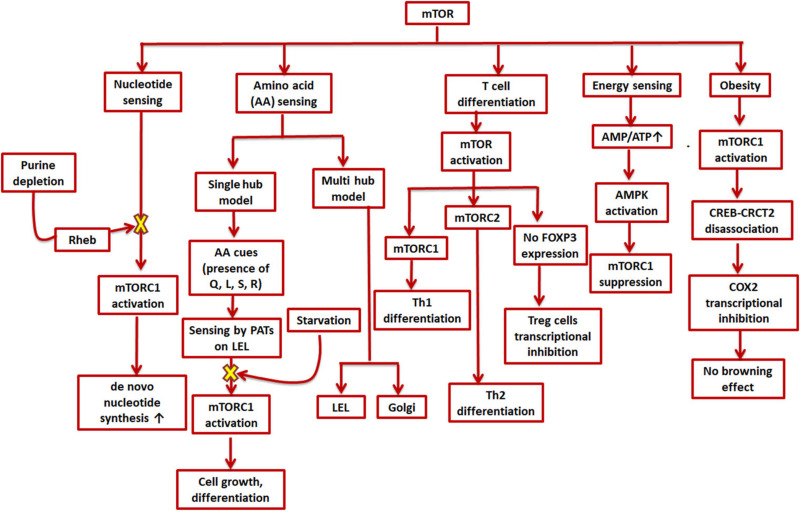
Role of mTOR in cell metabolism and signaling cascades. mTOR is involved in various environmental sensing and growth-related processes in the cell. mTORC1 stimulates *de novo* synthesis of nucleotides as well as responds to amino acid availability by promoting cell growth and differentiation; mTOR furthers helper T-cell differentiation while impeding regulatory T-cell differentiation; a higher AMP/ATP ratio can suppress mTORC1 and thus inhibit energy expending processes; and mTORC1 inhibits transcription of COX-2 which promotes obesity.

Along with growth factors, cellular energy levels, and oxygen levels, mTOR is also involved in sensing nutrients like amino acids and integrating these multitude of signals to produce a response according to the cell’s need to further grow or differentiate (Figure 2; Howie et al., 2014; Goberdhan et al., 2016). Two models have been suggested to describe amino acid sensing by mTORC1 and the subsequent effector responses: a single hub, comprising of a single mTORC1 collecting all the micro-environmental cues to produce a variety of outputs; and a multi-hub model, where two different mTORC1 function in different parts of the cell (Jewell et al., 2015; Fan et al., 2016; Goberdhan et al., 2016). Many amino acid transporters, such as members of the proton-assisted transporters (PATs) family, are localized on the late endosomal and lysosomal (LEL) surfaces (Goberdhan et al., 2005; Goberdhan, 2010; Figure 2). Apart from the LELs, other possible amino acid sensing locations via mTORC1 have also been suggested such as the Golgi complex, supporting the multi-hub model of sensing. The entire process of amino acid sensing by mTORC1 has been divided into a priming step (sensitization of mTORC1 for the final activation) and an actual activation step. L-Glutamine, L-glycine, L-glutamic acid, L-serine, and L-arginine are some of the amino acids involved in priming whereas activation is majorly carried out by L-leucine and to some extent by L-methionine, L-isoleucine, and L-valine. L-Cysteine mostly impedes the priming step. Some of these amino acids (leucine, glutamine, serine, and arginine) are known to aid in autophagy inhibition by activating mTORC1 (Shafei et al., 2017). As previously mentioned, autophagy dysfunction is a key player in AD development (Saxton and Sabatini, 2017). Moreover, amino acids as well as enzymes metabolizing them in AD patients are also known to deter from their normal levels (Shafei et al., 2017). Lower levels of branched chain amino acids like valine, especially in later stages of life, have been associated with a higher risk of AD (Tynkkynen et al., 2018). Cysteine has also been directly linked to AD and other neurodegenerative diseases as plasma levels of AD patients were found to have higher cysteine and sulfate ratio early in the morning. Higher cysteine implies higher thiol concentration in the cell, which could hinder with the functioning, confirmation, and synthesis of protein (Heafield et al., 1990). Glutamate is also known to regulate mTORC1via glutamate dehydrogenase (GDH) and human branched chain aminotransferase (hBCAT) enzymes. Interestingly, glutamate, which is a neurotransmitter involved in excitatory functions, has also been found to be toxic and damaging to neurons when present in higher concentrations (Schubert and Piasecki, 2001).

Analogous to amino acid sensing, mTORC1 is also involved in nucleotide sensing in a cell (Hoxhaj et al., 2017). As mTORC1 promotes ribosome biogenesis, 60% of which consists of rRNA, it was found to boost *de novo* nucleotide (both purine and pyrimidine) synthesis (Ben-Sahra et al., 2013; Figure 2). The pathway for *de novo* pyrimidine synthesis has been reported in an adult human brain, but whether or not mTORC1 has any role to play in it is not yet known. Moreover, the expression levels of several genes involved in synthesis of mRNAs (such as dihydroorotate dehydrogenase and uridine-cytidine kinase 2) for both the *de novo* as well as salvage pathways were found to be modified in case of AD patients (Pesini et al., 2019). The depletion of purines, specifically adenylates, was found to inhibit mTORC1 via the GTPase-activating protein (GAP) activity of TSC (Tuberous sclerosis complex) (Hoxhaj et al., 2017). The same study also reported mTORC1 inhibition on longer periods of guanylate-depletion due to degradation of Rheb. The TSC complex helps in maintaining Rheb in a GDP-bound state thereby inhibiting mTORC1 (Dibble et al., 2012). However, mTORC1 is also reported to be inhibited by guanylate depletion due to binding of Rheb-GTP and farnesylation (Emmanuel et al., 2017; Figure 2). Supplementation of uridine and other nutrients has positive effects in AD therapy (Engelborghs et al., 2014). In fact, uridine is also a part of the recipe for a rather popular medical supplement called Souvenaid^®^, known to support synaptic generation and function as a part of AD therapy (Ritchie et al., 2014).

The nutrient microenvironment inside a cell and sensing by mTOR is also closely related to T cell regulation, activation, and differentiation. While mTOR activation is needed for effector T cell proliferation and differentiation, it inhibits FOXP3 expression, the master transcription factor for regulatory T cells (Sauer et al., 2008; Delgoffe et al., 2010). Regulatory T cells are reported to be upregulated in an AD patient’s blood (Sommer et al., 2017). It was also shown that mTORC1 promotes selective Th1 differentiation while mTORC2 Th2 differentiation (Lee et al., 2010). The potential role of adaptive immune cells in AD, however, is still unclear with reports of both an enhanced pro- and anti-inflammatory response (Sommer et al., 2017; Figure 2).

The COX-2/PG axis, in addition to its role in tissue inflammation, also mediates insulin secretion in adipose tissue and differentiation of adipocytes (Fjaere et al., 2014). Epidemiological studies have linked diabetes and obesity with dementia (Ferreira et al., 2018). In fact, a lot of recent studies have highlighted the common cellular pathways underlying both diabetes and AD, thereby coining the term “type-3-diabetes” or “brain diabetes” to refer to AD (Xu et al., 2019). Anti-diabetic drugs are being explored as potential candidates of anti-AD therapy (De Felice, 2013). PGs have been reported to exert both pro- as well as anti-obesogenic effects (Madsen et al., 2008). Beige adipocytes play important roles in counteracting obesity and other related disorders like diabetes and cardiovascular diseases (Boström et al., 2012; Harms and Seale, 2013; Cohen et al., 2014; X. Zhang et al., 2018). The inter-communications between adipose tissue and CNS puts obese people at a higher risk of developing cognitive and other mental disorders (Forny-Germano et al., 2019). Interestingly, mTORC1 is also hyperactivated in adipose tissues of obese rodents and its enhanced expression in fact gives rise to adiposity as well as obesity (Um et al., 2004; Khamzina et al., 2005; Polak et al., 2008; Laplante and Sabatini, 2012). Most groups have reported that mTORC1 is inhibitory for thermogenesis and “browning effect” (conversion of white adipose tissue to brown/beige adipocytes), which help tackle obesity by inducing enhanced energy consumption processes (M. Um et al., 2004; Polak et al., 2008; Liu et al., 2014; Wada et al., 2016). Nevertheless, there have been a few studies which have shown that inactivation of mTORC1 also compromises thermogenesis (D. Liu et al., 2016; Tran et al., 2016). While exposure to cold is also known to induce browning effect in mammals, it has been found to modify pathways involved in AD in a study carried out in mice models (Xu et al., 2019). Since mTOR and COX-2 have been found to operate together directly or indirectly in various other disorders or conditions, it can also be speculated that they may affect each other in this particular case. In one such study, mTORC1 inactivation in the mice adipose tissue causes COX-2/PG pathway induction, sending off a paracrine signal to initiate browning effect (Zhang et al., 2018), whereas when mTORC1 is activated, it phosphorylates a serine residue (Ser^136^) in CREB-regulated transcription coactivator 2, preventing its efficient association with CREB and subsequently CREB binding with COX-2 promoter (Zhang et al., 2018). As CREB binding enhances COX-2 promoter activation (Reddy et al., 2000; Yang and Bleich, 2004), mTORC1 activation eventually leads to transcriptional inhibition of COX-2, thereby preventing beige adipogenesis (Figure 2).

## COX-2 and mTOR in Osmoprotection

**FIGURE 3:**
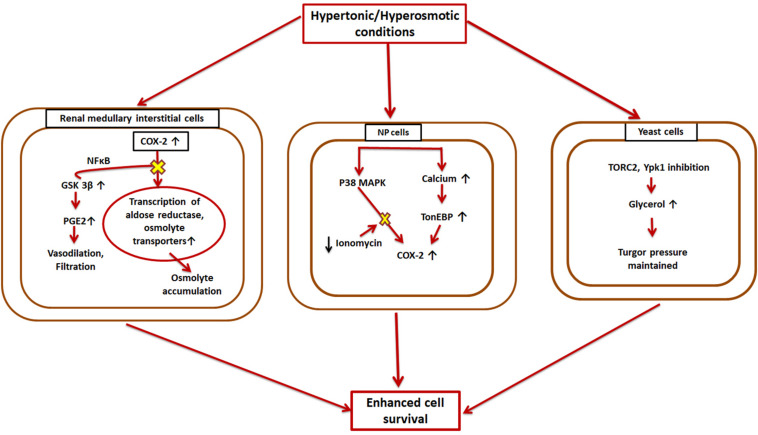
Hyperosmotic induction of COX-2 and mTOR. COX-2 is overexpressed in the reno-medullary interstitial cells in a hyperosmotic environment. This leads to higher transcriptional rates of osmolyte transporters, and thus the cell can accumulate large amounts of osmolytes to aid in its survival, while GSK-3β opposes this pathway; Nucleus pulposus (NP) cells also cope with hypertonic conditions by overexpressing COX-2 via two different cascades-TonEBP and MAPK; Yeast cells exhibit higher levels of TORC2 under hypertonic conditions resulting in higher glycerol concentrations which eventually reestablishes cell turgor pressure.

For normal cell functioning it is essential that the cellular volume, electrolytes, and other solute concentrations be maintained. Changes in the extracellular tonicity results in accompanying changes in the intracellular environment to ensure the cell maintains its normal volume (Macknight, 1988). The kidneys are one of those mammalian organs wherein cells are subjected to extreme concentrations of solutes like urea and NaCl (Burg et al., 2007). However, many diseases are known to be caused by the hyperosmotic stresses faced by non-renal tissues (Brocker et al., 2012). Cells deal with these conditions using certain small organic molecules, called osmolytes, that are dissolved in the cellular as well as extracellular fluids (Wijayasinghe et al., 2017). These organic osmolytes have been found to be accumulated in the renal medullary cells in situations of osmotic stress (Wijayasinghe et al., 2017). COX-2 and mTOR are involved in different kinds of osmoprotective pathways in different cell types (Figure 3).

Both COX-1 and COX-2 are expressed in different parts of the kidney (Y. Harris et al., 1994; Guan et al., 1997) with different regulatory mechanisms. While COX-2 exhibits expression based on the surrounding tonicity, COX-1 does not follow any such pattern (Briggs, 1999). Thus, there were speculations that COX-2 might have an osmoprotective function in the kidneys. Treatment with NSAIDs and COX-2 specific inhibitors in the reno-medullary interstitial (RMI) cells reduces osmolyte accumulation induced by hypertonic conditions in these cells and affects their survival (Moeckel et al., 2003). It was found that COX-2 causes this effect by transcriptional regulation of osmolyte transporters. Later on, it was also shown that GSK-3β kinase is a key player in this process and affects COX-2 via a signaling pathway involving NF-κB (Rao et al., 2004). The increased hypertonic conditions upregulate GSK-3β activity in the RMI cells, which increases their apoptotic rate (Rao et al., 2004). Thus, GSK-3β works as an antagonist of COX-2. TonEBP/NFAT5, a master transcription factor of osmoprotective genes (Favale et al., 2007), has also been found to enhance COX-2 mRNA levels in osmotically stressed RMI cells (Favale et al., 2009). MAPK family members and Src kinases are other known enzymes that are implicated in hypertonicity-induced COX-2 regulation (Burg et al., 2007; Yang et al., 2000; Figure 3).

Another part of the body that experiences higher and a constantly changing osmolarity is the inner core of vertebral disc, called nucleus pulposus (NP) (Choi et al., 2018). Therefore, the NP cells have mechanisms to adapt to these hyperosmotic microenvironments, which involve up-regulation of COX-2. This upregulation is intracellular calcium-dependent, but rather than the calceneurin signaling pathway, it occurs through TonEBP (Choi et al., 2018). It was found that TonEBP overexpression resulted in enhanced COX-2 promoter activity while its silencing resulted in diminished COX-2 promoter activity (Choi et al., 2018). TonEBP silencing also causes COX-2 enzyme levels to fall in iso-osmotic conditions (Choi et al., 2018). Additionally, p38 MAPK pathway also induced elevated expression of COX-2 under hyperosmotic conditions in NP cells. However, ionomycin treatment nullified this effect (Choi et al., 2018). This COX-2 overexpression eventually aids in (NP) cell survival under osmotic stress (Figure 3).

Additionally, in case of yeasts, mTORC2 (referred to as TORC2 in this case) has been demonstrated to be involved in osmotic-homeostasis maintenance (Eltschinger and Loewith, 2016; Figure 3). Ypk1 and Ypk2, homologs of Akt, are two important downstream substrates of TORC2. Hypertonic conditions inhibit TORC2 and Ypk1, which eventually results in increased glycerol concentrations in the cell and reestablishes the turgor pressure.

Osmolytes are reported to play corrective roles in many human pathologies caused by protein misfolding, although nothing specifically for AD (Kim et al., 2006). Meanwhile, a typical AD brain is marked by misfolded proteins that keep getting accumulated and result in autophagic and lysosomal dysfunction as well as amyloid and tau aggregate formation (Efeyan et al., 2013). In fact, hypertonicity is one of the many causes of protein damage in cells (Hahr, 2015).

## COX-2 Modulation of mTOR

**FIGURE 4:**
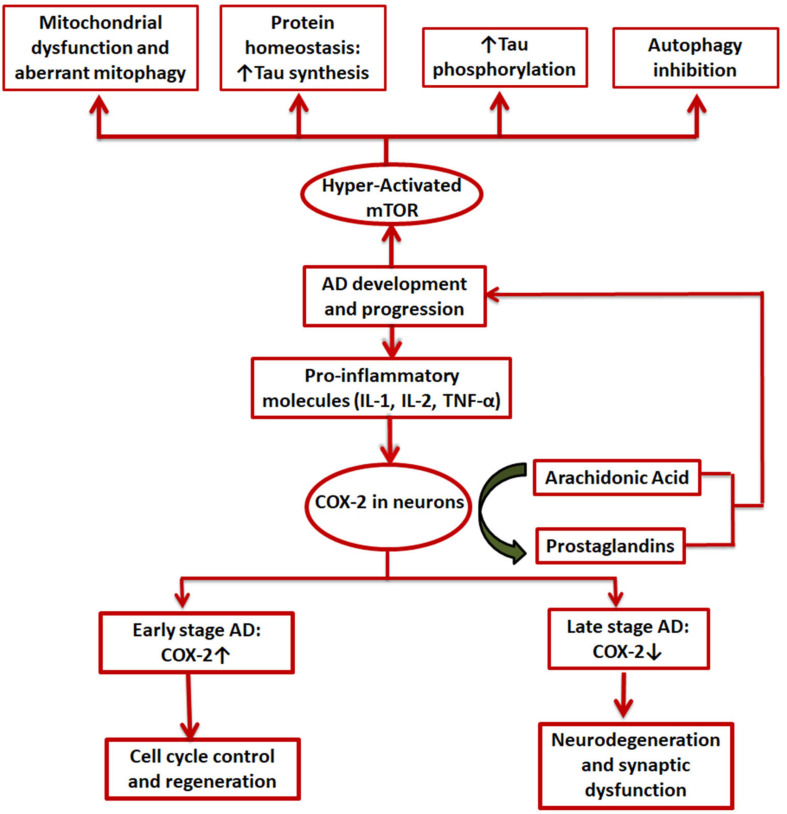
Involvement of COX-2 and mTOR in AD. Pro-inflammatory molecules induce COX-2 expression in certain brain cells. It is upregulated in the initial stages of AD and is expected to be involved in cell cycle control and regenerative pathways while in the later stages a decline in COX-2 levels is seen, mainly due to degeneration of neurons by the disease; mTOR, on the other hand, is always hyperactivated in case of AD and exerts its effects in mainly 4 ways: mitochondrial dysfunction, aberrant proteostasis, tau hyperphosphorylation, and inhibition of autophagy.

Both COX-2 and mTOR have been extensively studied individually and in combination with other speculated regulators of AD in the disease’s progression (Hoozemans et al., 2008; Pei and Hugon, 2008; Tang et al., 2015; Thomas et al., 2016; Mueed et al., 2019). The exact role COX-2 plays in the various stages of the disease is still debated, with reports claiming both beneficial as well as detrimental effects of the enzyme in the various AD associated pathologies (Minghetti, 2004; Hoozemans et al., 2008). However, mTOR is mostly known to be hyperactivated in AD and causes excessive phosphorylation of tau protein and formation of Aβ plaques and NFTs (Figure 4). Both have been implicated in development of synaptic plasticity under physiological conditions (Minghetti, 2004; Papadopoli et al., 2019). Interconnected signaling and working of COX-2 and mTOR has been the subject of many studies in the case of various types of cancer (Chuang et al., 2019(H. Lipskar et al., 2009; Rouzer and Marnett, 2009; Li H. et al., 2016), but no such studies have yet been conducted for AD. Both cancer and AD have a faulty DNA damage repair mechanism at their core that causes uninhibited growth and neuronal loss, respectively (Behrens et al., 2009). Rapamycin has been found to exert anti-tumor and anti-angiogenic effects by inhibiting neovascularization (Guba et al., 2002; Phung et al., 2006). It can do this via either an mTOR-dependent or independent pathway. In case of the mTOR-independent pathway, the phosphorylation of downstream effectors of mTOR remains unchanged and other molecules including COX-2 are instead involved (Jung et al., 2003; Lipskar et al., 2009). Moreover, cancer and AD have been shown to manifest an inverse relationship via many cellular pathways and signaling molecules such as p53 and cAMP and hormones like estrogen, growth factors, neutrophins, etc (Shafi, 2016).

In yet another study carried out on endometrial cancer (EMC) on mouse models and human cell lines, it was established that both COX-2 and mTORC1 work cooperatively to reduce tumor load and exacerbate the cancer. This common signaling pathway also involved Akt as both COX-2 and mTORC1 are downstream targets of Akt (Gadducci et al., 2008; Jae et al., 2009; Slomovitz and Coleman, 2012; Figure 4). Although these studies have been limited to mostly cancer models, similar pathways could exist in case of AD as well. If the presence of such a connection between COX-2 and mTOR could be established in AD, it could be exploited as a therapeutic strategy.

As mentioned in previous sections, mTOR controls T-cell activation and innate immune responses. mTOR is also known to modulate COX-2 expression in immune reactions. An *in vitro* study conducted on enhancement of immunomodulatory effects of human bone marrow mesenchymal stem cells showed elevated levels of COX-2 and higher phosphorylation of kinases, GSK-3b, and Akt, upon mTOR inhibition by short-term (4 h) rapamycin treatment (Wang et al., 2017; Figure 4). However, COX-2 is significantly downregulated on longer rapamycin treatments. PGE2, which plays important roles in immunosuppression by inhibition of NK (natural killer) cell cytotoxicity, dendritic cell maturation, and T cell proliferation (Spaggiari et al., 2008; Wang et al., 2017), was also found to be upregulated (Figure 4). Apart from COX-2 being overexpressed during inflammatory reactions including AD and cancers, it is also known to resist apoptosis (Tsujii and DuBois, 1995). Similar to COX-2, mTOR is also thought to be involved in apoptosis and activation of cell cycle in post mitotic neurons (Pei and Hugon, 2008; Figure 4). Thus, NSAIDs and selective COX-2 inhibitors induce apoptosis. Two pathways have been suggested for this: stimulation of ceramide (a death signal) production by availability of higher amounts of AA (Chan et al., 1998), and downregulation of Bcl-2 (X. Liu et al., 1998; Sheng et al., 1998). Alternatively, a selective COX-2 inhibitor, celecoxib, is thought to induce apoptosis by selectively blocking Akt pathway (Hsu et al., 2000). However, the mechanism behind this remains elusive as celecoxib does not significantly affect the major kinase, PI-3K, or the phosphatase, protein phosphatase 2A, involved in the activation of Akt (Hsu et al., 2000). Interestingly, this might indicate another possible pathway by which COX-2 modulates mTOR signaling as Akt is one of the major upstream regulators of mTOR.

## COX-2 and mTOR-Based Therapies

As discussed earlier, NSAIDs were found to be associated with a lower vulnerability to AD due to lowering levels of Aβ42 as well as reducing inflammation. A lot of studies have been conducted in this regard exploring different aspects of the disease development and progression. Various therapeutic approaches for AD have also been reviewed previously (Mueed et al., 2019).

It was found that selective COX-2 inhibitors could restore long term potentiating which was lost due to incubation with external Aβ42 in studies carried out in rat hippocampal slices, while inhibition of only COX-1 failed to produce any such effects (Kotilinek et al., 2008). The same study also reported regarding restoration of memory in Tg2576 mice over-expressing APP by both selective COX-2 inhibitors as well as non-selective NSAIDs. However, these beneficial effects of COX-2 inhibition were lost on addition of exogenous PGE2 but not by inflammatory cytokines like IL-1β, TNF-α, or even Aβ. Thus, the authors proposed an alternative mechanism of NSAID action on AD: preventing PGE2 response at synapses due to blockage of COX-2.

As previously stated, mTOR is known to be hyperactivated in an AD brain and exerts its effects by affecting key cellular processes such as nucleotide synthesis, protein synthesis, and modifications, mitochondrial dynamics, lipid metabolism and the cellular autophagic system. Therapies against AD and other neurodegenerative disorders have thus also targeted mTOR. Inhibition of mTOR using rapamycin and its analogs has shown reduction in the amounts of amyloid and tau deposits in early stage-AD-affected brains of mice and improved their cognitive abilities (Spilman et al., 2010; Majumder et al., 2011). In fact, humans who were administered with a rapamycin analog, everolimus, in order to elicit immunosuppression after a heart transplant also showed better memory skills than the control group (Papadopoli et al., 2019). mTOR overexpression has been routinely linked to intracellular tau accumulation and translocation. Its inhibition via rapamycin or silencing by mutation leads to lower tau levels in the cell (Tang et al., 2015). This has implications across various tauopathies and presents a potential therapeutic approach. mTOR inactivation has proved to be helpful in reducing microglial-inflammatory responses in the brain. Some of these studies have also shown a possible COX involvement in the process as rapamycin treated microglia also resulted in lower levels of COX-2 (Dello Russo et al., 2013).

COX-2 is also implicated in a variety of cancers (Hwang et al., 1998; Pandey et al., 2008; Greenhough et al., 2009; Alexanian et al., 2014; Mattsson et al., 2015) and its inhibition can also be used as one of the ways for cancer therapy. Tuberous sclerosis complex (TSC) caused as a result of mutations in any of the two tumor suppressor genes, *TSC1* and *TSC2*, leads to formation of localized tumor masses in multiple organs (Borkowska et al., 2011; Kwiatkowski and Manning, 2014; Schepis, 2016). mTORC1 is known to be hyperactivated by loss of TSC2 in TSC tumors (Moavero et al., 2015; Li H. et al., 2016). This leads to downregulation of COX-2 activity via activation of STAT3 (signal transducer and activator of transcription 3). It was also found that IL-6 is a downstream target of COX-2 in cells with loss-of-Tsc2 mutation and thus reduced activity of COX-2 inhibits IL-6 which limits cellular proliferation (Li H. et al., 2016). Rapamycin and celecoxib, together, were found to be most useful against TSC in Tsc2 negative cells, than either individually (Li H. et al., 2016).

## Conclusion and Future Perspectives

COX-2 is involved in diverse cellular functions in the human body such as PG synthesis, insulin secretion, adipogenesis, CNS autonomic regulation, etc., and thus, any structural or functional aberrations in the enzyme will have varied repercussions across many pathways. Many inflammatory and neoplastic disorders are related to COX-2 expression levels and activity. Similarly, mTOR affects central cellular activities such as growth, transcription, and translation. It has also been found to play a vital role in inflammatory and age-related diseases. COX-2 and mTOR operate in a number of overlapping pathways such as nutrient sensing, obesity, apoptosis, immune reactions, osmoprotection, etc.

Alzheimer’s is one of the world’s leading types of dementia and a neuro-inflammatory disease. COX-2 has been classically implicated in AD and much research has been done to develop therapies against AD based on blocking or deleting COX-2. However, it has been known for some time now that neither the cause of AD nor the role COX-2 plays in its occurrence and progression is as it was traditionally considered. There have been many views rejecting the amyloid as well as tau hypotheses to be at the heart of AD. Also, COX-2 is now thought to have pleiotropic effects in the brain and AD pathophysiology as the enzyme exhibits different expression levels during different stages of the disease with elevated levels in the early stages and a gradual fall later on. Moreover, other molecular targets of NSAIDs rather than the COX enzymes are being considered as possible key players in AD. Thus, in order to effectively tackle AD, it has to be considered in its entirety and not as isolated underlying pathways.

mTOR entered the AD picture as a molecule of interest quite recently but it has since been well established in AD pathology. It is supposedly overexpressed in AD, promotes amyloid- as well as tau-deposition, and is also involved in their targeting to various cell organelles to a certain extent. Inhibition of mTOR *in vitro* and *in vivo* models of AD has demonstrated improved memory and other cognitive abilities which are compromised in the disease.

The main aim of this review was to explore the biphasic nature of relationship between COX-2 and mTOR in key cellular processes, some of which have direct roles in AD progression. This suggests a very strong possibility that their relationship could be exploited in order to develop an effective counter for AD. Moreover, their actions have already been found to be inter-dependent in the case of a number of cancers, and anti-cancer therapies targeting the two of them are underway. However, dedicated studies in this direction in the case of AD are still, to the best of our knowledge, non-existent. The possible future prospective in this direction would include exploring in detail selected targets of interest, such as Akt, and all their associated pathways which may provide specific answers as to if and how COX-2 and mTOR co-operate in AD and if drugs targeting either or both of these would be helpful in combating the disease.

## Author Contributions

AT wrote the manuscript. NP made the background of the manuscript and finalized the manuscript. MK gave valuable comments and help in writing the manuscript. All authors contributed to the article and approved the submitted version.

## Conflict of Interest

The authors declare that the research was conducted in the absence of any commercial or financial relationships that could be construed as a potential conflict of interest.
